# Physics-driven Spatiotemporal Regularization for High-dimensional Predictive Modeling: A Novel Approach to Solve the Inverse ECG Problem

**DOI:** 10.1038/srep39012

**Published:** 2016-12-14

**Authors:** Bing Yao, Hui Yang

**Affiliations:** 1 Complex Systems Monitoring, Modeling and Control Laboratory, The Pennsylvania State University, University Park, 16802, USA

## Abstract

This paper presents a novel physics-driven spatiotemporal regularization (STRE) method for high-dimensional predictive modeling in complex healthcare systems. This model not only captures the physics-based interrelationship between time-varying explanatory and response variables that are distributed in the space, but also addresses the spatial and temporal regularizations to improve the prediction performance. The STRE model is implemented to predict the time-varying distribution of electric potentials on the heart surface based on the electrocardiogram (ECG) data from the distributed sensor network placed on the body surface. The model performance is evaluated and validated in both a simulated two-sphere geometry and a realistic torso-heart geometry. Experimental results show that the STRE model significantly outperforms other regularization models that are widely used in current practice such as Tikhonov zero-order, Tikhonov first-order and L1 first-order regularization methods.

Linear regression is a widely used approach for modeling the relationship between explanatory variables ***x***’s and response variable ***y*** by the linear function, ***y*** = ***Rx*** + ***ε***, in which ***R*** is a parameter matrix characterizing the model details. Linear regression has widespread applications in various fields such as engineering, healthcare, economics and social science, for predictive modeling, experimental design, or system optimization. Regression parameters are often estimated based on the static data set of explanatory and response variables. However, rapid advancement of distributed sensing and imaging technology brings the proliferation of high-dimensional spatiotemporal data, i.e., ***y*** = ***y**(s, t*) and ***x*** = ***x**(s, t*) in healthcare systems. Traditional regression is not generally applicable for predictive modeling in these complex structured systems.

For example, Fig. 1 shows the distribution of electric potentials ***y**(s, t*) acquired by the ECG sensor network placed on the body surface, also named body surface potential mapping (BSPM)^1^,^2^. Medical scientists call for the estimation of electric potentials ***x**(s, t*) on the heart surface from BSPM ***y**(s, t*) so as to investigate cardiac pathological activities (e.g., tissue damages in the heart)^3^,^4^,^5^,^6^. However, spatiotemporally varying data and complex torso-heart geometries defy traditional regression modeling and regularization methods.

In general, high-dimensional predictive modeling (i.e., ***y**(s, t*) = ***Rx**(s, t*) + ***ε***) poses several challenges including

(1) **Physics-based derivation of parameter matrix** ***R***: Traditional regression modeling estimates parameter matrix ***R*** based on the readily available data set of [***x**, **y***]. However, distributed sensing or imaging of spatiotemporal systems provides only the surface profiles ***y**(s, t*) such as BSPMs. It is often difficult to directly measure heart-surface potential mappings ***x**(s, t*). As such, inferring ***x**(s, t*) needs a better knowledge of parameter matrix ***R***. Fortunately, physical laws define the mechanisms of electrical propagation from the heart to the body surface. This, in turn, enables the derivation of parameter matrix ***R*** using physics-based principles (i.e., divergence theorem, Green’s theorem).

(2) **Ill-conditioned system**: Linear systems involving high-dimensional data ***y**(s, t*) and ***x**(s, t*) are commonly ill-conditioned. This is partly caused by unobserved ***x**(s, t*), and partly due to the fact that parameter matrix ***R*** is rank deficient (i.e., *rank(**R***) < *min*{*dim(**x***),*dim(**y***)}). The condition number of ***R*** (i.e., *cond(**R***) = ||***R***||||***R***^−1^||) is also shown to be large in high-dimensional predictive modeling (e.g., inverse ECG problems^7^,^8^). Moreover, the derivation of ***R*** depends, to a great extent, on deterministic physics-based principles and the numerical analysis of complex geometries but does not account for real-world uncertainties. Such uncertainties may be introduced by simplified physical assumptions, geometric variations, measurement noises and other extraneous factors. As a result, high-dimensional prediction models cannot always match satisfactorily with data from real-world experiments.

(3) **Spatiotemporal regularization**: Ill-conditioned systems make the prediction more sensitive to noise factors (e.g., ***ε***) and approximation errors in parameter matrix ***R***. For example, measurement noises can potentially cause a small change Δ***y*** in the observed data ***y**(s, t*). Considering the estimation of ***x*** changes to ***x*** + Δ***x***, we will have the changes in the solution expressed as 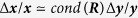. Because of the large condition number *cond(**R***), the pseudo-inverse solution of 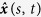 may be completely different. As such, there is an urgent need to develop new statistical approaches that leverage physics-based principles and observed data to account for uncertainties and tackle the ill-conditioned problems. Although ***x**(s, t*) and ***y**(s, t*) are spatially distributed and dynamically evolving over time, they have spatial and temporal correlations. Very little has been done to develop new spatial regularization methods that handle approximation errors through spatial correlations of dynamic profiles on the complex geometry (e.g., the heart surface), as well as new temporal regularization methods to increase model robustness to measurement noises and other uncertainty factors.

This paper presents a new spatiotemporal regularization model to tackle these research challenges and address ill-condtioned problems in high-dimensional predictive modeling. Our contributions in the present investigation are as follows:

(1) High-dimensional systems involve complex geometries, which challenge the derivation of parameter matrix ***R***. We developed realistic models of torso-heart geometries, numerically discretized them with the boundary element method, and then utilized physical laws (i.e., divergence theorem and Green’s theorem) to derive the parameter matrix.

(2) As physics-based models are deterministic and do not account for real-world uncertainties, we developed a physical-statistical approach that integrates physics-derived parameter matrix ***R*** with a spatiotemporal regularization (STRE) method to build the high-dimensional prediction model. This approach leverages data from actual experiments to improve spatial and temporal regularity of the solutions, thereby making the final prediction closer to reality.

(3) The proposed STRE model involves quadratic programming and high-dimensional data, which cannot be solved analytically. Iterative algorithms are commonly used such as the multiplicative update method which, however, requires the nonnegative constraint of ***x**(s, t*). As such, they are not generally applicable because the electric field involves both positive and negative potentials. We developed a new method of dipole multiplicative update, which is inspired by the dipole assumption in electrodynamic physics. This new idea overcomes the drawbacks of existing multiplicative update methods, and provides a generalized approach to solve spatiotemporal regularization problems.

(4) Few, if any, previous works focused on both spatial and temporal regularizations in inverse and forward ECG problems. We evaluated and validated the proposed STRE model in simulation as well as a real-world case study to map electric potentials from the body to the heart surface. Experimental results show that our method not only effectively tackles the ill-conditioned problems in high-dimensional predictive modeling, but also outperforms those regularization models widely used in current practice (i.e., Tikhonov zero-order, Tikhonov first-order and L1 first-order regularization methods). This research work provides a new and effective approach to investigate disease-altered electric potentials from the body to the heart surface.

The remainder of this paper is organized as follows: Section II introduces the research background. Section III presents our research methodology. Section IV describes the experimental design. Experimental results are shown in section V. Section VI concludes this paper.

## Research Background

### Ill-conditioned systems

The high-dimensional predictive model, ***y**(s, t*) = ***Rx**(s, t*) + ***ε***, where ***x**(s, t*) and ***y**(s, t*) are spatiotemporal data, is generally ill-conditioned. For example, the inverse ECG problem in healthcare (i.e., mapping the potential distribution on the heart surface from the body surface)^7^,^8^ is ill-conditioned. The condition number of the parameter matrix ***R*** (i.e., *cond(**R***) = ||***R***||||***R***^−1^||) is a measure of relative sensitivity of the solution ***x**(s, t*) to the observed data ***y**(s, t*) (i.e., 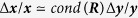), which is shown to be large in prediction models that involve high-dimensional data and complex structured systems. The large value of *cond(**R***) indicates that the prediction model is highly sensitive to changes in ***y**(s, t*). The pseudo-inverse solution of 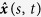 in traditional regression methods (i.e., 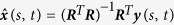) is unreliable and sensitive to uncertainty factors. Therefore, additional physical or statistical constraints are required to guarantee the norm of the solution to be regular and increase the reliability of the high-dimensional prediction model.

### Regularization Methods

Statistical regularization models such as Tikhonov and L1 regularization methods^7^,^8^,^9^,^10^ were proposed to address the ill-conditioned parameter matrix ***R***, increase the model reliability and improve the prediction accuracy.

The objective function of Tikhonov regularization is formulated as


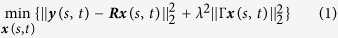


while the L1 regularization is formulated as


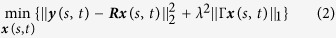


where ||·||_2_ and ||·||_1_ denote the L2- and L1-norm, respectively, *λ* is the regularization parameter, and Γ represents the mathematical operator constraining ***x**(s, t*). Note that Γ is the identity matrix in zero-order Tikhonov and L1 regularization methods (also known as ridge regression^11^ and LASSO^12^ in statistics), which directly penalize the magnitude of the estimator.

Zero-order regularization is effective to shrink unreliable components of the estimator and achieve sparse solutions for high-dimensional predictive modeling. However, they are limited in the ability to handle measurement noises or approximation errors in ill-conditioned systems. Therefore, first-order regularization methods were proposed to address such limitations by constraining the gradient of the solution ***x**(s, t*). Note that Γ is a discretized gradient operator in the first-order regularization methods. One of the most commonly used gradient operators is a bidiagonal matrix^9^,^10^ expressed as


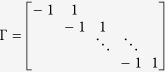


which is a central-difference approximation for the first-order derivative. However, this approximation does not account for the complex geometries of space-time dynamic systems, and is only effective for one-dimensional data. Most of previous works aligned ***x**(s, t*) in one column as {***x**(s*_1_|*t*), ***x**(s*_2_|*t*), …, ***x**(s*_*N*_|*t*)}^*T*^, and then applied the bidiagonal gradient matrix. Note that the alignment of spatiotemporal data in one column is not an effective way (maybe even incorrect) to compute the spatial gradients. As such, regularization results are not as satisfactory as expected.

In the inverse ECG problem, another commonly used gradient operator is the normal derivative-operator of the potential distribution on the heart surface, Γ***x**(s, t*) = ∂***x**(s, t*)/∂***n***, where ***x**(s, t*) denotes the dynamic potential distribution on the heart surface and ***n*** denotes the surface-normal vector^7^,^8^. However, this operator only includes the normal derivative of ***x**(s, t*), but ignores the gradient component on the the heart surface (i.e., ∂***x**(s, t*)/∂***τ***, where ***τ*** denotes the surface-tangent vector) and does not take into account the spatial correlations between adjacent regions. It is worth mentioning that spatiotemporal data from distributed sensing and imaging are generally spatially distributed and have spatial correlations^13^,^14^. In the existing first-order regularization methods, the gradient operator Γ does not account for the spatial correlations or complex geometries of space-time systems. Thus, it is imperative to develop new regularization models to handle the approximation errors and improve the spatial regularity of the solution in high-dimensional predictive modeling.

In addition, space-time systems are dynamically varying over time and have temporal correlations. For example, the human heart is a typical spatiotemporal system with cardiac electrical activities dynamically varying in both space and time^15^,^16^. Messnarz *et al*.^17^ proposed a spatiotemporal approach to reconstruct cardiac electric potentials. Spatial correlation is addressed by a surface gradient of the solution that is approximated using a symmetric matrix. The temporal constraint is formulated on the assumption that electric potentials on the heart surface are monotonically nondecreasing during the depolarization phase. However, the geometry of heart surface is highly complex, and thus a symmetric matrix tends to be limited in the ability to approximate the surface gradient. Moreover, the nondecreasing assumption in the temporal constraint may not be generally applicable to high-dimensional predictive modeling. Thus, there is an urgent need to design a novel spatiotemporal regularization method with the ability to effectively improve the spatial and temporal regularities in space-time systems.

## Research Methodology

**Figure 2:**
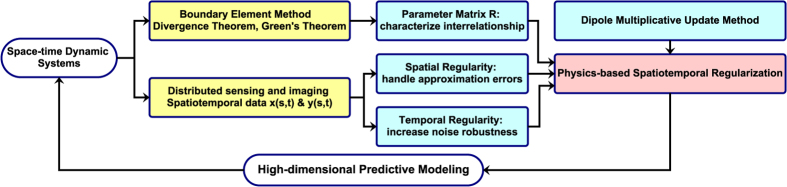
Flowchart of research methodology.

As shown in Fig. 2, modern industries are increasingly investing in distributed sensing and imaging technology to cope with complexity in space-time dynamic systems. This brings large amount of spatiotemporal data (e.g., potential mappings in cardiology). This section presents a new physics-driven spatiotemporal regularization (STRE) approach for high-dimensional predictive modeling. First, we derive the parameter matrix ***R*** by integrating the boundary element method with divergence theorem and Green’s theorem. Second, we investigate the spatial regularization that handles approximation errors through spatial correlations of dynamic profiles on the complex geometry (i.e., heart surface), as well as the temporal regularization to increase model robustness to measurement noises. Finally, we develop a new generalized method of dipole multiplicative update to solve the objective function of the proposed STRE model.

### Physics-based Derivation of Parameter Matrix *R*

The observed data ***y**(s, t*) are generally obtained from the surface of a complex structured system such as BSPMs. Inferring the internal dynamic variable ***x**(s, t*) (e.g., electric potential distributions on the heart surface) of these systems depends on the high-dimensional predictive modeling


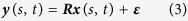


where ***R*** is the parameter matrix characterizing the interrelationship between ***x**(s, t*) and ***y**(s, t*).

In the human body system, the heart represents the bioelectric source, and the torso is modeled as a homogeneous and isotropic volume conductor whose boundary consists of body surface *S*_*B*_ and heart surface *S*_*H*_^18^,^19^. Electric potentials ***x**(s, t*) on the heart surface and ***y**(s, t*) on the body surface are related by the Laplace’s equations derived from physics-based principles (i.e., divergence theorem and Green’s theorem). Solving for the parameter matrix ***R*** involves tackling this Laplace’s equation and calculating complex surface integrations, which are difficult to solve analytically in realistic torso-heart geometry. Thus, boundary element method (BEM)^20^,^21^ is implemented to discretize *S*_*B*_ and *S*_*H*_ into triangle meshes, and divide the surface integrals into a series of numerical integrations over the triangle elements. Thus, the parameter matrix ***R*** is expressed as^18^,^19^


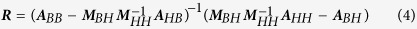


where the coefficient matrices, ***A***'s and ***M***'s depend entirely on the torso-heart geometry. The rows of ***A***_*BB*_, ***A***_*BH*_ and ***M***_*BH*_ correspond to the locations of different nodes on the body triangle-mesh *S*_*B*_. Similarly, the rows of ***A***_*HH*_, ***A***_*HB*_ and ***M***_*HH*_ represent the locations of different nodes on the heart triangle-mesh *S*_*H*_. The different columns of all the matrices correspond to locations of triangle elements on the surface of integration.

However, inferring ***x**(s, t*) in complex structured systems is an ill-conditioned problem, because the parameter matrix ***R*** is often with a large condition-number^7^,^8^. Moreover, several assumptions have been made when deriving matrix ***R***. For examples, the human body is modeled as a homogeneous volume conductor, and geometrical variations over time are assumed to be negligible. These assumptions may not hold true in real-world situations and will introduce uncertainties when predicting ***x**(s, t*)^22^,^23^. Thus, obtaining a numerically robust solution of high-dimensional predictive modeling calls for the integration of physics-based principles with new statistical regularization methods.

### Spatial and Temporal Regularization

The spatiotemporal data acquired by distributed sensing and imaging systems are generally distributed in the space and have spatial correlations. In existing regularization methods, the constraint operator Γ or the penalty term does not account for the spatial correlations or the geometries of complex systems, but rather align the mesh nodes in one column or take the normal derivative operator. As such, they are limited in the ability to improve the spatial regularity. In this investigation, we propose to define the constraint operator Γ to be a spatial Laplacian operator Δ_*s*_ to overcome the drawbacks in existing methods.

**Figure 3:**
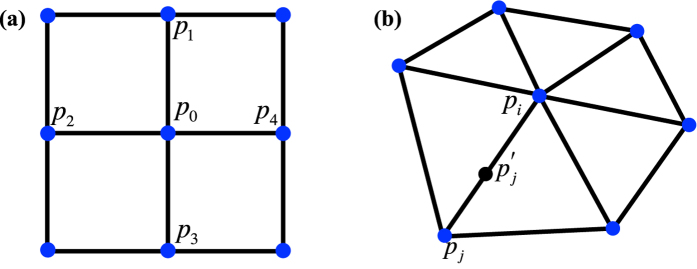
(**a**) 2D square lattice; (**b**) 3D triangle mesh.

The matrix Δ_*s*_ is computed by determining the Laplacian at each mesh node. In a two-dimensional square lattice with a lattice constant *d* as shown in Fig. 3(a), *x*_*i*_ denotes the value of dynamic variable ***x**(s, t*) at node *p*_*i*_ = (*u*_*i*_, *v*_*i*_), where (*u*_*i*_, *v*_*i*_) are location coordinates. According to Taylor’s theory, *x*_*i*_ is approximated as the sum of *x*_0_ and its derivatives at node *p*_0_ = (*u*_0_, *v*_0_):


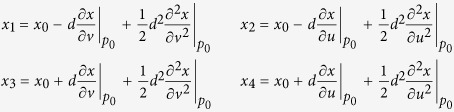


Adding the above four equations yields


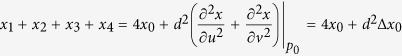


Thus, the Laplacian of *x*_0_ at node *p*_0_ is expressed as


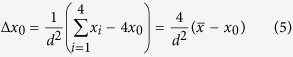


where 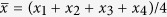. Finally, the surface Laplacian of this square lattice is


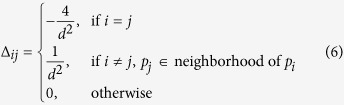


However, real-world geometries are complex and are generally discretized into irregularly triangulated meshes using the boundary element method^20^,^21^ as shown in Fig. 3(b). Unlike the 2D square lattice, the Euclidean distance between different pairs of nodes is not a constant on the 3D triangle mesh. Thus, we estimate the Laplacian at each mesh node by linear interpolation. In this 3D triangle mesh, *x*_*t*_(*i*) denotes the value of dynamic variable ***x**(s, t*) at node *p*_*i*_ at time *t*, and *d*_*ij*_ is the distance between *p*_*i*_ and *p*_*j*_. Using linear interpolation, the value 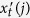 at the location 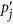 which is along the edge of *p*_*i*_ and *p*_*j*_, and 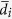 away from *p*_*i*_, as shown in Fig. 3(b), is expressed as


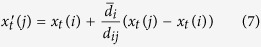


where 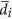 is the average of *d*_*ij*_’s over the neighbor nodes *p*_*j*_’s of *p*_*i*_, and these neighbors *p*_*j*_’s are the vertices of the triangles that include *p*_*i*_ as one of the vertices. Thus, the Laplacian of *x*_*t*_(*i*) at *p*_*i*_ in a 3D triangle mesh is defined as


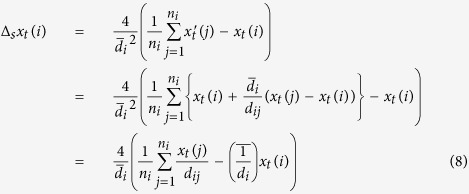


where *n*_*i*_ is the number of neighbor nodes *p*_*j*_’s of *p*_*i*_; 
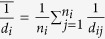
, denotes the average of 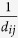 over these *p*_*j*_’s. According to Eq. (8), we define the elements of the Laplacian matrix Δ_*s*_ as


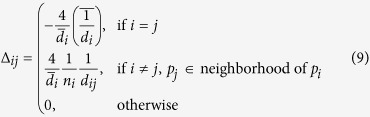


Therefore, the spatial regularity of three-dimensional triangle mesh at node *p*_*i*_ is defined as


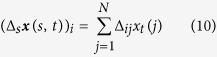


where *N* is the total number of mesh nodes.

In addition, spatiotemporal data ***x**(s, t*) and ***y**(s, t*) are dynamically evolving over time and have temporal correlations. However, few, if any, previous works have effectively dealt with the temporal regularization for high-dimensional predictive modeling in space-time systems (i.e., ***y**(s, t*) = ***Rx**(s, t*) + ***ε***, the two-body dynamic prediction problem). Therefore, we propose to define the temporal regularity as


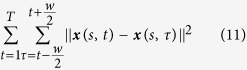


where *T* denotes the length of the overall time span of the spatiotemporal data, and *w* is a time window. Temporal correlation is stronger when two time points are close to teach other, and electric potentials at two time points that are far away from each other tend to have bigger differences. Therefore, the time window *w* is often chosen to be a small number. Adding the temporal constraints in Eq. (11) to our regularization model is conducive to increase the model robustness to measurement noises in the time domain.

### Spatiotemporal Regularization (STRE) Model

Combining the parameter matrix, spatial and temporal regularization as described in previous subsections, we formulate our STRE model by the following objective function


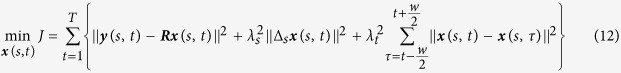


where *λ*_*s*_ and *λ*_*t*_ are the spatial and temporal regularization parameters, which can be chosen by the L-curve method^24^ or cross validation. By adding both the spatial and temporal regularization into the objective function, the proposed model will not only handle the approximation errors in ***R***, but also increase the model robustness to measurement noises in the time domain. Therefore, it is expected that the proposed STRE method will greatly improve the performance of high-dimensional predictive modeling in space-time systems.

This objective function involves both spatial and temporal correlations, and is difficult to be solved analytically. Iterative algorithms are commonly used such as the multiplicative update method which, however, requires nonnegative constraint of ***x**(s, t*)^25^,^26^. As such, they are not generally applicable because both negative and positive electric potentials exist on the heart or body surface. Here, we develop a dipole multiplicative update method to solve the proposed STRE model, inspired by the dipole assumption in electrodynamic physics. In this method, ***x***_*t*_ is split into its positive part 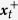 and negative part 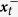, which are defined as 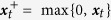 and 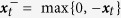. Thus, ***x***_*t*_ can be denoted as 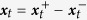. To simplify notation, we use ***y***_*t*_ and ***x***_*t*_ to denote ***y**(s, t*) and ***x**(s, t*) here and later on. Then the term that only depends on *vectx*_t_ in the objective function becomes


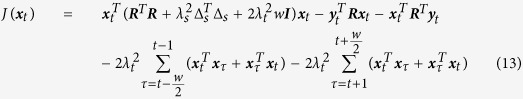


where ***I*** is an identity matrix whose dimension is the same as the Laplacian matrix Δ_*s*_. We substitute 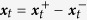 into Eq. (13) and define


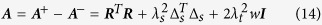



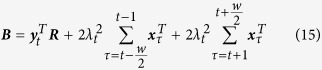


where matrix ***A***^+^ and ***A***^−^ are the positive and negative parts of matrix ***A***, whose definition is similar to that of 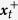 or 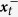. We then obtain the update rules shown in Table 1. See the detailed proof in Appendix B.

## Experimental Design

In the present investigation, the proposed STRE model is implemented to predict the time-varying distribution of electric potentials on the heart surface from real-world sensor data of electric potentials on the body surface. The model performance is evaluated and validated in both a simulated two-sphere geometry and a realistic torso-heart geometry.

### Simulation Studies in a Two-sphere Geometry

**Figure 4:**
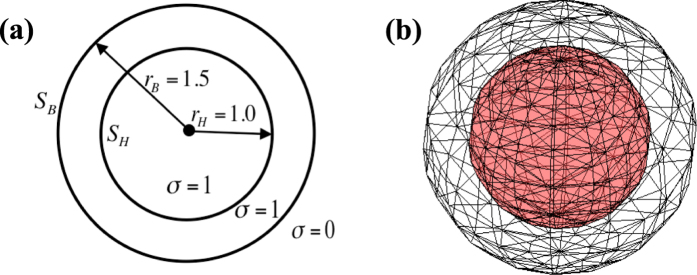
(**a**) Parameters of the two-sphere geometry; (**b**) Each sphere is triangulated with 184 nodes and 364 triangles.

Figure 4 shows the simulated two-sphere geometry that is formed by two concentric spheres. Each sphere is triangulated with 364 triangles and 184 nodes, which generates a 184 × 184 parameter matrix ***R***. A time-varying three-dimensional current dipole **p**(**t**) = (*p*_*x*_(*t*), *p*_*y*_(*t*), *p*_*z*_(*t*)) is placed at the center of the two-sphere geometry, which is defined as


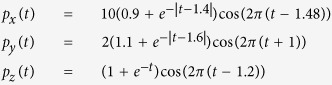


where time *t* ranges from 0 ms to 300 ms. Thus, the dynamic distributions of electric potentials on the inner surface ***x**(s, t*) and outer surface ***y**(s, t*) are calculated analytically by the equations^27^:


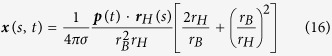



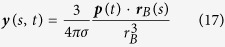


where *σ* = 1 is the electric conductivity inside the outer sphere, ***r***_*H*_(*s*) and ***r***_*B*_(*s*) denote the location vectors from the center to the inner and outer spheres, respectively, and *r*_*H*_ = 1.0 and *r*_*B*_ = 1.5 are the radii of the two spheres.

The proposed STRE model is implemented to predict the electric potentials 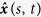 on the inner sphere based on electric potentials ***y**(s, t*) on the outer sphere calculated by Eq. (17). Regularization parameters *λ*_*s*_ = 0.015 and *λ*_*t*_ = 0.5 are chosen by the L-curve method^24^, and time window *w* is specified to be 2. In our simulation studies, Gaussian noises with mean zero and variance 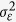 (i.e., 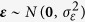) are added to ***y**(s, t*). Five different noise levels (i.e., 10%, 20%, 30%, 40%, 50%) are added at each time, which correspond to noises with standard deviations *σ*_*ε*_ = 0.1, 0.2, 0.3, 0.4, 0.5, respectively. At each noise level, the predicted potentials on the inner sphere will be compared with the true data (i.e., reference potentials) calculated by Eq. (16).

### Real-world Case Studies in a Realistic Torso-heart Geometry

**Figure 5:**
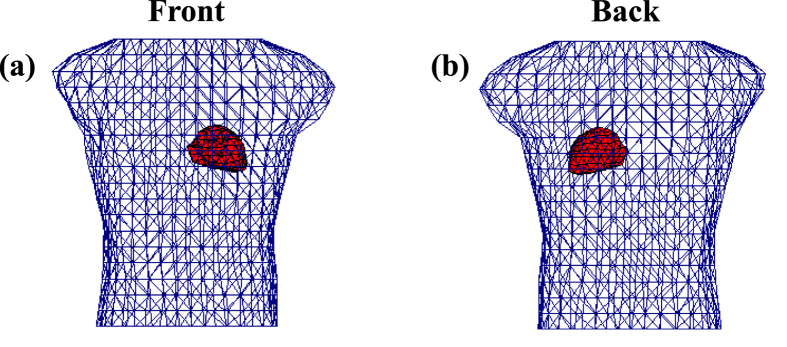
(**a**) Front and (**b**) back views of the realistic torso-heart geometry. The heart surface is triangulated with 257 nodes and 510 triangles and the torso surface is triangulated with 771 nodes and 1538 triangles.

Furthermore, we conduct experiments in the realistic torso-heart geometry, as shown in Fig. 5. The data of electric potentials (whose recording length is a complete cycle of heartbeat and *t* ranges from 0ms to 1000 ms) on the heart and body surfaces, and the torso-heart geometry are obtained from the Center for Integrative Biomedical Computing (CIBC) at the University of Utah^28^. In this torso-heart geometry, the heart surface consists of 257 nodes and 510 triangles, while the torso surface is formed by 771 nodes and 1538 triangles. The BSPM ***y**(s, t*) are acquired from 367 sensors, which are located at 367 nodes on the body surface. Thus, a 367 × 257 parameter matrix ***R*** is generated. The STRE model is implemented to predict the potential distribution 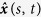 on the heart surface from the BSPM ***y**(s, t*). Regularization parameters *λ*_*s*_ = 2.0 and *λ*_*t*_ = 0.005 are chosen by the L-curve method^24^, and the time window *w* is specified to be 2.

Similarly, five different noise levels (i.e., 0.6%, 1.3%, 6.3%, 12.6%, 25.3%) are added to the electric potentials on the body surface ***y**(s, t*) to simulate the real-world uncertainties in this torso-heart geometry. The five noise levels are with standard deviations *σ*_*ε*_ = 0.005, 0.01, 0.05, 0.1, 0.2, respectively. The estimated electric potentials on the heart surface from high-dimensional predictive modeling will be benchmarked with real-world sensor data of reference potentials.

### Performance Evaluation

The performance metric, relative error (RE), is used to evaluate the model performance, i.e.,


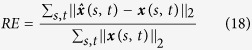


where 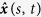 and ***x**(s, t*) denote the estimator and reference results, respectively. The performance of our STRE model is benchmarked with Tikhonov zero-order (Tikh_0th), Tikhonov first-order (Tikh_1st) and L1 first-order (L1_1st) regularization methods. In these first-order regularization methods, the matrix Γ is defined as the normal derivative operator of the electric potentials on the inner surface^7^,^8^. The methods to solve Tikhonov and L1 regularizations are described in Appendix A.

## Results and Discussions

### Experimental Results in the Two-sphere Geometry

**Figure 6:**
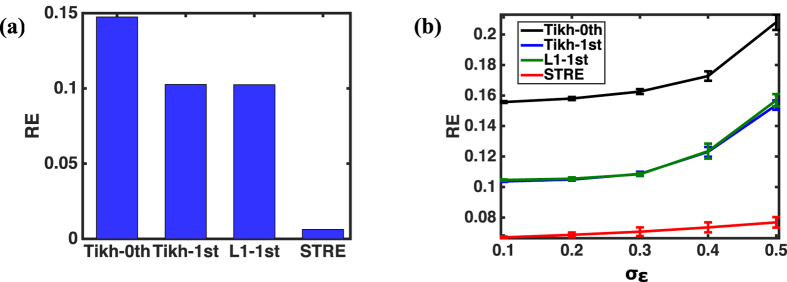
(**a**) The comparisons of relative error (RE) between the proposed STRE model and other regularization methods (i.e., Tikhonov zero-order, Tikhonov first-order and L1 first-order methods) in the two-sphere geometry when there is no noise on the potential map ***y**(s, t*) of the outer sphere; (**b**) The comparisons of RE between the proposed STRE model and other regularization methods (i.e., Thikhonov zero-order, Tikhonov first-order and L1 first-order methods) for different noise levels *σ*_*ε*_ = 0.1, 0.2, 0.3, 0.4, 0.5 on the potential map ***y**(s, t*) of the outer sphere.

Figure 6(a) shows the comparisons of relative error (RE) between the proposed STRE model and other regularization methods (i.e., Tikhonov zero-order, Tikhonov first-order and L1 first-order methods) in the two-sphere geometry, when there is no noise on the potential map ***y**(s, t*) of the outer sphere. Note that the proposed STRE model yields the RE of 0.006, which is significantly smaller than that obtained from Tikh_0th, Tikh_1st and L1_1st, which are 0.1475, 0.1026, and 0.1025, respectively.

Figure 6(b) shows the variations of RE for different regularization methods with respect to the noise level added to the potential map ***y**(s, t*) of the outer sphere. In the present investigation, we replicated the experiment 20 times for each noise level, and thus the resulted RE is shown with a corresponding error bar (i.e., the standard deviation of RE). When the noise level increases from *σ*_*ε*_ = 0.1 to *σ*_*ε*_ = 0.5, the RE monotonically increases for all the methods. Specifically, the RE increases from (0.0670 ± 0.00057) to (0.0769 ± 0.0034) for the proposed STRE model, from (0.1557 ± 0.00058) to (0.2080 ± 0.005) for Tikh_0th, from (0.1037 ± 0.00031) to (0.1538 ± 0.0031) for Tikh_1st, and from (0.1046 ± 0.0004) to (0.1569 ± 0.0041) for L1_1st. Notably, the STRE model yields the smallest RE for all noise levels, and achieves the slowest increase of RE with respect to the noise level among various regularization methods.

**Figure 7:**
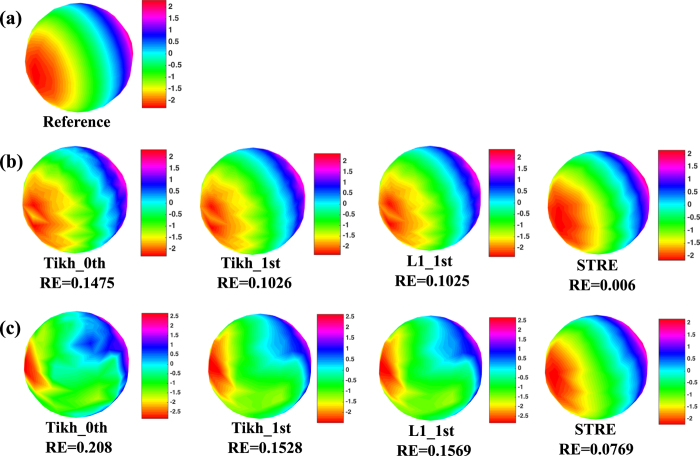
(**a**) Reference potential mapping on the inner sphere ***x**(s, t*), *t* = 150 *ms*, in the two-sphere geometry. (**b**) The comparisons of predicted potential mapping on the inner sphere ***x**(s, t*), *t* = 150 *ms*, between the STRE model and other regularization methods (i.e., Thikhonov zero-order, Tikhonov first-order and L1 first-order methods) when there is no noise on the potential map ***y**(s, t*) of the outer sphere. (**c**) The comparisons of predicted potential mapping on the inner sphere ***x**(s, t*), *t* = 150 *ms*, between the STRE model and other regularization methods (i.e., Thikhonov zero-order, Tikhonov first-order and L1 first-order methods) with the noise level *σ*_*ε*_ = 0.5 on the potential map ***y**(s, t*) of the outer sphere.

Furthermore, Fig. 7(a) shows the reference mapping of the true potential distribution on the inner sphere calculated by Eq. (16), whose value ranges from −2.5 *mV* to 2.5 *mV*. Note that the potential distribution on the inner sphere is dynamically varying over time, and Fig. (7) illustrates the mapping at *t* = 150 *ms*. Figure 7(b) shows the predicted potential mappings on the inner sphere by different methods when there is no noise on the potential map ***y**(s, t*) of the outer sphere. Note that the predicted potential mapping by the STRE yields a smaller RE of 0.006 compared to that of Tikh_0th (i.e., 0.1475), Tikh_1st (i.e., 0.1026) and L1_1st (i.e., 0.1025), which achieves the best performance to predict the reference potential mapping shown in Fig. 7(a). Figure 7(c) shows the predicted potential mappings on the inner sphere by different methods with noise level *σ*_*ε*_ = 0.5 in ***y**(s, t*) of the outer sphere. Notably, the predicted potential mappings by Tikh_0th, Tikh_1st and L1_1st under this noise level show different color patterns from the results under the condition of no noise, and their RE’s are 0.208, 0.1528 and 0.1569, respectively. However, the predicted mapping by the proposed STRE model closely preserves the color patterns of the results with no noise, and yields the smallest RE of 0.0769.

As shown in Figs 6 and 7, the proposed STRE model achieves the best performance among these regularization methods when predicting the dynamic potential distribution on the inner sphere in this two-sphere geometry. The model performance of Tikh_0th is the worst among all the methods, which is due to the fact that zero-order regularization method does not account for the spatial or temporal correlations in the data, but rather penalizes the magnitude of the estimator to achieve sparse solutions. The RE’s of Tikh_1st and L1_1st are around the same level, which is because the gradient operators of these two regularization methods are the same (i.e., the normal derivative operator). In the regular spherical geometry, the normal derivative operator does account for the spatial correlations to some extent in this simulation study, and thus these two first-order methods perform better than Tikh_0th. However, the temporal correlations are not well considered in Tikh_1st or L1_1st, and their RE’s are higher compared to that of the proposed STRE model. Experimental results show that the proposed STRE model achieves the smallest RE and increases the model robustness to measurement noises by improving both the spatial and temporal regularities of the solution.

### Experimental Results in the Realistic Torso-heart Geometry

**Figure 8:**
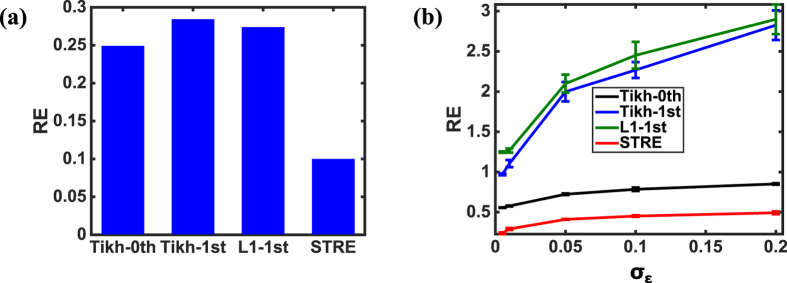
(**a**) The comparisons of relative error (RE) between the proposed STRE model and other regularization methods (i.e., Thikhonov zero-order, Tikhonov first-order and L1 first-order methods) in the realistic torso-heart geometry when there is no extra noise on the potential map ***y**(s, t*) of the body surface; (**b**) The comparisons of RE between the proposed STRE model and other regularization methods (i.e., Thikhonov zero-order, Tikhonov first-order and L1 first-order methods) for different noise levels *σ*_*ε*_ = 0.005, 0.01, 0.05, 0.1, 0.2 on the potential map ***y**(s, t*) of the body surface.

Figure 8(a) shows the comparisons of relative error (RE) between the proposed STRE model and other regularization methods (i.e., Tikhonov zero-order, Tikhonov first-order and L1 first-order methods) in the realistic torso-heart geometry, when there is no additional noise on the potential map ***y**(s, t*) of the body surface. In the present investigation, our STRE model yields a much smaller RE of 0.0997 compared to that of Tikh_0th (i.e., 0.2488), Tikh_1st (i.e., 0.2839) and L1_1st (i.e., 0.2735). Note that the RE’s of all the methods in this realistic torso-heart geometry are relatively bigger compared to the results in the simulated two-sphere geometry when no extra noise is added to ***y**(s, t*). This is mainly due to the fact that ***y**(s, t*) are real-world BSPM data with measurement noises and other uncertainty factors in the inverse ECG problem, while that in the simulated two-sphere geometry are clean data calculated analytically by Eq. (17).

Figure 8(b) shows the variations of RE with respect to the noise level for different regularization methods. Although there are already measurement noises in the sensor data of potential map ***y**(s, t*) on the body surface, we added different levels of noises to increase the real-world uncertainties on ***y**(s, t*). In the present investigation, we also replicated the experiment 20 times for each noise level, and thus each resulted RE is shown with a corresponding error bar (i.e., standard deviation of RE). When the noise level increases from *σ*_*ε*_ = 0.005 to *σ*_*ε*_ = 0.2, the RE monotonically increases for all the methods. Specifically, the RE increases from (0.2386 ± 0.0105) to (0.4933 ± 0.0175) for the proposed STRE model, from (0.5570 ± 0.0025) to (0.8521 ± 0.0086) for Tikh_0th, from (0.9720 ± 0.0115) to (2.8261 ± 0.1835) for Tikh_1st, and from (1.2481 ± 0.0082) to (2.8994 ± 0.1849) for L1_1st, respectively. It is worth mentioning that the RE’s increase dramatically when adding noises to ***y**(s, t*) on the body surface, compared to the results in the simulated two-sphere geometry. This is mainly due to the fact that the realistic torso-heart geometry is much more complex and irregular. As such, the resulted high-dimensional prediction model tends to be more sensitive to noises. Nevertheless, our STRE model yields the smallest RE for all noise levels, and achieves the slowest increase of RE with respect to the noise level among various regularization methods in this realistic torso-heart geometry.

**Figure 9:**
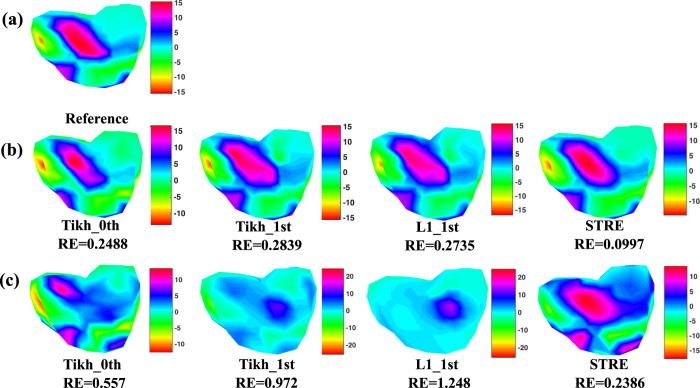
(**a**) Reference potential mapping on the heart surface *x(s, t*), *t* = 50 *ms*, in the realistic torso-heart geometry. (**b**) The comparisons of predicted potential mappings on the heart surface ***x**(s, t*), *t* = 50 *ms*, between the STRE model and other regularization methods (i.e., Thikhonov zero-order, Tikhonov first-order and L1 first-order methods) when there is no extra noise on the potential map ***y**(s, t*) of the body surface. (**c**) The comparisons of predicted potential mappings on the heart surface ***x**(s, t*), *t* = 50 *ms*, between the STRE model and other regularization methods (i.e., Thikhonov zero-order, Tikhonov first-order and L1 first-order methods) with the noise level *σ*_*ε*_ = 0.005 on the potential map ***y**(s, t*) of the body surface.

Furthermore, Fig. 9(a) shows the reference mappings of measured potential distribution on the heart surface, whose value ranges from −15 *mV* to 15 *mV*. Note that the potential distribution on the heart surface is dynamically varying over time, and Fig. (9) illustrates the heart-surface potential mapping when *t* = 50 *ms*. Figure 9(b) shows the predicted potential mappings on the heart surface by different methods, when there is no additional noise on the potential map ***y**(s, t*) of the body surface. Note that the proposed STRE yields the RE of 0.997, which is significantly smaller than that of Tikh_0th (i.e., 0.2488), Tikh_1st (i.e., 0.2839) and L1_1st (i.e., 0.2735), and yields the best performance to predict the reference potential mapping shown in Fig. 9(a). Figure 9(c) shows the predicted potential mappings by different methods with the noise level *σ*_*ε*_ = 0.005 in ***y**(s, t*) on the body surface. It is worth mentioning that the predicted potential mappings by Tikh_0th, Tikh_1st and L1_1st under this noise level show significantly different color patterns from Fig. 9(a) and (b). Their RE’s are 0.557, 0.927 and 1.248, respectively. However, the STRE model yields the smallest RE of 0.2386 and approximately preserves the color patterns in real-world data of potential mapping on the heart surface.

As shown in Figs 8 and 9, the proposed STRE model achieves the best performance among various regularization methods when predicting the dynamic potential distribution on the heart surface in this realistic torso-heart geometry. The inferior performance of Tikh_0th, Tikh_1st and L1_1st is due to the fact that they neither effectively address the spatial regularity in the inverse ECG problem nor take into account the temporal correlations of the space-time systems. It may be noted that the RE’s of Tikh_1st and L1_1st are higher than that of Tikh_0th, which is not the case in the simulated two-sphere geometry. This is because the realistic torso-heart geometry is more complex and irregular than the simulated two-sphere geometry. The normal derivative operator in Tikh_1st and L1_1st can address the spatial correlations to some extent in the regular two-sphere geometry, but will lead to incorrect approximations in the complex heart geometry. As such, this causes additional errors to the solution in the prediction model. The proposed STRE model effectively addresses both spatial and temporal regularities of the solution, thereby yielding the smallest RE and increasing the model robustness to measurement noises or real-world uncertainties.

## Conclusions

Advanced sensing and imaging technology lead to the proliferation of spatiotemporal data ***x**(s, t*) and ***y**(s, t*). This poses significant challenges for high-dimensional predictive modeling (i.e., ***y**(s, t*) = ***Rx**(s, t*) + ***ε***) in complex systems (e.g., solving the inverse ECG problem). First, inferring ***x**(s, t*) needs a better knowledge of parameter matrix ***R*** that characterizes the physics-based interrelationship between ***x**(s, t*) and ***y**(s, t*). Second, ill-conditioned systems make the predictions more sensitive to measurement noises and approximation errors in ***R***. Third, very little has been done to develop new spatial regularization methods that handle approximation errors, as well as new temporal regularization methods to increase model robustness to measurement noises. Thus, there is an urgent need to tackle these research challenges and address ill-conditioned problems in high-dimensional predictive modeling.

In this paper, we developed a physics-driven spatiotemporal regularization (STRE) model for predicting dynamic behaviors in space-time systems. First, we developed realistic models of torso-heart geometry, and utilized the boundary element method and physics-based principles (i.e., divergence theorem, Green’s theorem) to derive the parameter matrix ***R***. Second, we developed a physical-statistical approach that integrates physics-derived parameter matrix ***R*** with a spatiotemporal regularization method to build the high-dimensional predictive model. Third, we designed a new method of dipole multiplicative update, inspired by the dipole assumption in electrodynamic physics, to solve the generalized spatiotemporal regularization problems.

The proposed STRE model is implemented to predict potential distribution on the heart surface using BSPM data. The model performance is evaluated and validated in both a simulated two-sphere geometry and a realistic torso-heart geometry. Experimental results show that our method not only effectively tackles the ill-conditioned problems in high-dimensional predictive modeling, but also outperforms those regularization models widely used in current practice (i.e., Tikhonov zero-order, Tikhonov first-order and L1 first-order regularization methods). The present research work provides a new and effective approach to investigate disease-altered electric potentials on the heart surface in healthcare systems.

## Additional Information

**How to cite this article**: Yao, B. and Yang, H. Physics-driven Spatiotemporal Regularization for High-dimensional Predictive Modeling: A Novel Approach to Solve the Inverse ECG Problem. *Sci. Rep.* **6**, 39012; doi: 10.1038/srep39012 (2016).

**Publisher's note:** Springer Nature remains neutral with regard to jurisdictional claims in published maps and institutional affiliations.

## Supplementary Material

Supplementary Information

## Figures and Tables

**Figure 1:**
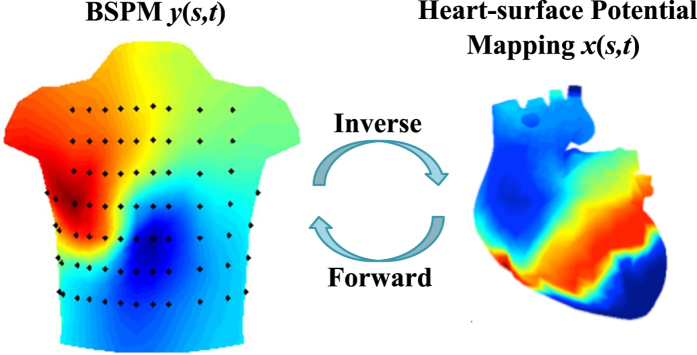
Spatiotemporal distribution of electrical potentials on the body and heart surfaces. (Note that black dots are ECG sensors placed on the body surface).

**Table 1:** The Proposed New Dipole Multiplicative Update Algorithm for STRE.

1:	Set constants *λ*_*s*_, *λ*_*t*_ and *w*.
2:	Initialize {***x***^+^} and {***x***^−^} as positive random matrices:
	whose columns (rows) denote different time points (different nodes on the heart surface)
3:	**Repeat**
4:	**for** *t* = 1, …, *T* **do**
	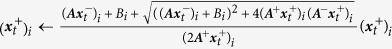
	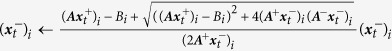
5:	**end for**
6:	**until convergence**
