# Supplementary material for: Physics-driven Spatiotemporal Regularization for High-dimensional Predictive Modeling: A Novel Approach to Solve the Inverse ECG Problem
Source: Sci Rep. 2016 Dec 14;6:39012. doi: 10.1038/srep39012 (PMC5155286; doi:10.1038/srep39012)
Supplement: Supplementary Information [file srep39012-s1.pdf]

Physics-driven Spatiotemporal Regularization for High-dimensional Predictive Modeling: A Novel Approach to Solve the Inverse ECG Problem

Bing Yao and Hui Yang*
Complex Systems Monitoring, Modeling and Control Laboratory
The Pennsylvania State University, University Park, 16802, USA
*Corresponding author: huy25@psu.edu

Supplementary Information for Scientific Reports

A. Tikhonov Regularization

The cost function of Tikhonov Regularization is

$$\begin{aligned} J_{Tikh} &= \|\boldsymbol{y} - \boldsymbol{R}\boldsymbol{x}\|_2^2 + \lambda^2 \|\Gamma \boldsymbol{x}\|_2^2 \\ &= \boldsymbol{x}^T(\lambda^2 \Gamma^T \Gamma + \boldsymbol{R}^T \boldsymbol{R})\boldsymbol{x} - \boldsymbol{x}^T \boldsymbol{R}^T \boldsymbol{y} - \boldsymbol{y}^T \boldsymbol{R}\boldsymbol{x} + \boldsymbol{y}^T \boldsymbol{y} \end{aligned} \tag{1}$$

In order to minimize the cost function, the derivative of J_{Tikh} is taken and set to zero

$$\frac{\partial J_{Tikh}}{\partial \boldsymbol{x}} = 2(\lambda^2 \Gamma^T \Gamma + \boldsymbol{R}^T \boldsymbol{R})\boldsymbol{x} - 2\boldsymbol{R}^T \boldsymbol{y} = 0 \tag{2}$$

Thus, the estimator of Tikhonov regularization is expressed as

$$\hat{\boldsymbol{x}} = (\lambda^2 \Gamma^T \Gamma + \boldsymbol{R}^T \boldsymbol{R})^{-1} \boldsymbol{R}^T \boldsymbol{y} \tag{3}$$

B. L1 Regularization

The cost function of the L1 regularization is

$$J_{L1} = \|\boldsymbol{y} - \boldsymbol{R}\boldsymbol{x}\|_2^2 + \lambda^2 \|\Gamma \boldsymbol{x}\|_1 \tag{4}$$

Due to the L1-norm, the cost function J_{L1} is non-differentiable and the estimator of the L1 regularization cannot be solved analytically. Thus, the lagged diffusivity iteration algorithm is implemented to solve L1 regularization [1]:

Initialize:

$$\hat{\boldsymbol{x}}^{(0)} = (\lambda^2 \Gamma^T \Gamma + \boldsymbol{R}^T \boldsymbol{R})^{-1} \boldsymbol{R}^T \boldsymbol{y} \tag{5}$$

Repeat for step $k = 1, 2, 3...$ until convergence:

$$W_\beta^k(\hat{\boldsymbol{x}}^{(k-1)}) = \frac{1}{2}\text{diag}[\frac{1}{\sqrt{|\Gamma \hat{\boldsymbol{x}}^{(k-1)}|_1^2 + \beta}}] \tag{6}$$

$$\hat{\boldsymbol{x}}^{(k)} = (\lambda^2 \Gamma^T W_\beta^k(\hat{\boldsymbol{x}}^{(k-1)})\Gamma + \boldsymbol{R}^T \boldsymbol{R})^{-1} \boldsymbol{R}^T \boldsymbol{y} \tag{7}$$

where $\beta = 10^{-5}$ is small positive number to guarantee that the denominator of each element in $W_{\beta}^{k}(\hat{\boldsymbol{x}}^{(k-1)})$ is nonzero.

C. The Proof of Dipole Multiplicative Update Rules

An auxiliary function $G(\{\boldsymbol{z}_t^+ - \boldsymbol{z}_t^-\}, \{\boldsymbol{x}_t^+ - \boldsymbol{x}_t^-\})$ is defined for the objective function of our STRE model, which satisfies: (1)$J(\{\boldsymbol{z}_t^+ - \boldsymbol{z}_t^-\}) \leq G(\{\boldsymbol{z}_t^+ - \boldsymbol{z}_t^-\}, \{\boldsymbol{x}_t^+ - \boldsymbol{x}_t^-\})$ and (2) $J(\{\boldsymbol{x}_t^+ - \boldsymbol{x}_t^-\}) = G(\{\boldsymbol{x}_t^+ - \boldsymbol{x}_t^-\}, \{\boldsymbol{x}_t^+ - \boldsymbol{x}_t^-\})$ for any positive vectors, $\boldsymbol{z}_t^+$, $\boldsymbol{z}_t^-$, $\boldsymbol{x}_t^+$ and $\boldsymbol{x}_t^-$. Then the update rule can be defined as $\{\boldsymbol{x}_t^+, \boldsymbol{x}_t^-\} = \arg\min_{\{\boldsymbol{z}_t^+, \boldsymbol{z}_t^-\}} G(\{\boldsymbol{z}_t^+ - \boldsymbol{z}_t^-\}, \{\boldsymbol{x}_t^+ - \boldsymbol{x}_t^-\})$, which does not increase the value of the objective function.

The objective function in terms of $\boldsymbol{z}_t$ is expressed as

$$\begin{aligned} J &= \sum_{t=1}^{T}\{\boldsymbol{z}_t^T\boldsymbol{A}^+\boldsymbol{z}_t - \boldsymbol{z}_t^T\boldsymbol{A}^-\boldsymbol{z}_t - \boldsymbol{B}\boldsymbol{z}_t - \boldsymbol{z}_t^T\boldsymbol{B}^T\} \\ &= \sum_{t=1}^{T}\{((\boldsymbol{z}_t^+)^T - (\boldsymbol{z}_t^-)^T)\boldsymbol{A}^+(\boldsymbol{z}_t^+ - \boldsymbol{z}_t^-) - ((\boldsymbol{z}_t^+)^T - (\boldsymbol{z}_t^-)^T)\boldsymbol{A}^-(\boldsymbol{z}_t^+ - \boldsymbol{z}_t^-) \\ &\quad -\boldsymbol{B}(\boldsymbol{z}_t^+ - \boldsymbol{z}_t^-) - ((\boldsymbol{z}_t^+)^T - (\boldsymbol{z}_t^-)^T)\boldsymbol{B}^T\} \\ &= \sum_{t=1}^{T}\{(\boldsymbol{z}_t^+)^T\boldsymbol{A}^+\boldsymbol{z}_t^+ + (\boldsymbol{z}_t^-)^T\boldsymbol{A}^+\boldsymbol{z}_t^- - (\boldsymbol{z}_t^+)^T\boldsymbol{A}^-\boldsymbol{z}_t^+ - (\boldsymbol{z}_t^-)^T\boldsymbol{A}^-\boldsymbol{z}_t^- \\ &\quad -((\boldsymbol{z}_t^-)^T\boldsymbol{A}^+ + \boldsymbol{B})\boldsymbol{z}_t^+ - (\boldsymbol{z}_t^+)^T(\boldsymbol{B}^T + \boldsymbol{A}^+\boldsymbol{z}_t^-) \\ &\quad +((\boldsymbol{z}_t^+)^T\boldsymbol{A}^- - \boldsymbol{B})\boldsymbol{z}_t^- - (\boldsymbol{z}_t^-)^T(\boldsymbol{B}^T - \boldsymbol{A}^-\boldsymbol{z}_t^+)\} \end{aligned} \tag{8}$$

where $\boldsymbol{A}$ and $\boldsymbol{B}$ are defined in Eq. (27) and Eq. (28). For any positive vectors $\mathbf{u}$ and $\mathbf{v}$, the following expressions are proven to hold.

$$\mathbf{u^T A^+ u} \leq \sum_i \frac{(\mathbf{A^+ v})_i}{v_i} u_i^2 \tag{9}$$

$$\mathbf{u^T A^- u} \geq \sum_{ij} A_{ij}^- v_i v_j (1 + log\frac{u_i u_j}{v_i v_j}) \tag{10}$$

Therefore, the auxiliary function in terms of $\boldsymbol{z}_t$ and $\boldsymbol{x}_t$ can be written as

$$\begin{aligned} G &= \sum_{t=1}^{T}\{\sum_i \frac{(\boldsymbol{A}^+\boldsymbol{x}_t^+)_i}{(\boldsymbol{x}_t^+)_i}(\boldsymbol{z}_t^+)_i^2 + \sum_i \frac{(\boldsymbol{A}^+\boldsymbol{x}_t^-)_i}{(\boldsymbol{x}_t^-)_i}(\boldsymbol{z}_t^-)_i^2 - \sum_{ij} A_{ij}^-(x_t^+)_i(x_t^+)_j(1 + log\frac{(z_t^+)_i(z_t^+)_j}{(x_t^+)_i(x_t^+)_j}) \\ &\quad -\sum_{ij} A_{ij}^-(x_t^-)_i(x_t^-)_j(1 + log\frac{(z_t^-)_i(z_t^-)_j}{(x_t^-)_i(x_t^-)_j}) - \sum_i 2\{((\boldsymbol{x}_t^-)^T\boldsymbol{A})_i(\boldsymbol{z}_t^+)_i + ((\boldsymbol{x}_t^+)^T\boldsymbol{A})_i(\boldsymbol{z}_t^-)_i \\ &\quad -2B_i((z_t^+)_i - (z_t^-)_i)\}\} \end{aligned} \tag{11}$$

Taking the derivatives of G_t with respect to $\boldsymbol{z}_t^+$ and $\boldsymbol{z}_t^-$ and setting them to zero, we have

$$\frac{\partial G}{\partial (z_t^+)_i} = 2\frac{(\boldsymbol{A}^+\boldsymbol{x}_t^+)_i}{(\boldsymbol{x}_t^+)_i}(z_t^+)_i - 2\frac{(\boldsymbol{A}^-\boldsymbol{x}_t^+)_i(\boldsymbol{x}_t^+)_i}{(z_t^+)_i} - 2(B_i + (\boldsymbol{A}\boldsymbol{x}_t^-)_i) = 0 \tag{12}$$

$$\frac{\partial G}{\partial (z_t^-)_i} = 2\frac{(\boldsymbol{A}^+\boldsymbol{x}_t^-)_i}{(\boldsymbol{x}_t^-)_i}(z_t^-)_i - 2\frac{(\boldsymbol{A}^-\boldsymbol{x}_t^-)_i(\boldsymbol{x}_t^-)_i}{(z_t^-)_i} - 2(-B_i + (\boldsymbol{A}\boldsymbol{x}_t^+)_i) = 0 \tag{13}$$

Solving the above two equations, we obtain

$$(x_t^+)_i \leftarrow (z_t^+)_i = \frac{(\boldsymbol{A}\boldsymbol{x}_t^-)_i + B_i + \sqrt{((\boldsymbol{A}\boldsymbol{x}_t^-)_i + B_i)^2 + 4(\boldsymbol{A}^+\boldsymbol{x}_t^+)_i(\boldsymbol{A}^-\boldsymbol{x}_t^+)_i}}{2(\boldsymbol{A}^+\boldsymbol{x}_t^+)_i}(x_t^+)_i \quad (14)$$

$$(x_t^-)_i \leftarrow (z_t^-)_i = \frac{(\boldsymbol{A}\boldsymbol{x}_t^+)_i - B_i + \sqrt{((\boldsymbol{A}\boldsymbol{x}_t^+)_i - B_i)^2 + 4(\boldsymbol{A}^+\boldsymbol{x}_t^-)_i(\boldsymbol{A}^-\boldsymbol{x}_t^-)_i}}{2(\boldsymbol{A}^+\boldsymbol{x}_t^-)_i}(x_t^-)_i \quad (15)$$

which leads to the algorithm of dipole multiplicative update shown in table 1.

References

[1] S. Ghosh and Y. Rudy, "Application of l1-norm regularization to epicardial potential solution of the inverse electrocardiography problem," *Annals of Biomedical Engineering*, vol. 37, no. 5, pp. 902–912, 2009.
